# Supplementary material for: Optimal adaptive cancer therapy based on evolutionary game theory
Source: PLoS One. 2025 Apr 23;20(4):e0320677. doi: 10.1371/journal.pone.0320677 (PMC12017572; doi:10.1371/journal.pone.0320677)
Supplement: Attachment — Submitted filename: Response to Reviewers.pdf [file pone.0320677.s001.pdf]

Dear Dr. Kavallaris and respected reviewers:

Thank you for reviewing our manuscript titled "Optimal Adaptive Cancer Therapy Based on Evolutionary Game Theory" and for providing valuable feedback. We have carefully considered all the reviewers' suggestions and revised the manuscript accordingly. Below is our point-by-point response to the reviewers' comments:

Reviewer 1

Major Comments:

Comment 1:

- **Reviewer's Comment:** Can a weighting factor be introduced between the two terms in Equation 10? When t_f approaches infinity, does the first term in the objective function lose its role in the control problem?
- **Authors' Response:** The issue you raised is crucial, and we fully agree. In the original objective functional, the integral term may dominate as time increases, potentially diminishing the role of the first term. To address this, we have introduced a weighting coefficient in the revised version, modifying the functional as:

$$\min J(x,m) = \lambda \psi(T(t_f)) + \int_0^{t_f} \eta m(t) dt.$$

Here, the weighting coefficient λ and η are selected based on practical requirements to balance the contributions of both terms. This adjustment prevents the integral term from dominating over long time horizons while preserving the regulatory role of the terminal term $\psi(T(t_f))$.

- **Revision Location:** Lines 113–114, Lines 149–151， Lines 154–155,Equations 10, 11, 17,24 and 27.

Comment 2:

- **Reviewer's Comment:** Please elaborate in the Discussion section on why the optimized adaptive therapy strategy is superior to other treatment strategies.
- **Authors' Response:** Thank you for the suggestion. We have added a discussion comparing the advantages of our optimal adaptive therapy strategy with other methods, including those proposed by West et al., citing relevant literature.
- **Revision Location:** Lines 444–496.

Minor Comments:

Comment 1:

- **Reviewer's Comment:** A citation is missing in Line 246.
- **Authors' Response:** Thank you for pointing this out. We have added the appropriate reference.
- **Revision Location:** Line 247.

Comment 2:

- **Reviewer's Comment:** Clarify the meaning of "environmental factors" in Line 247.
- **Authors' Response:** "Environmental factors" refer to patient-specific differences influencing cancer cell dynamics, such as genetic mutations, organ function, immune status, and drug metabolism. These factors lead to heterogeneous responses to therapy, as validated by clinical data from Virginia et al. (https://doi.org/10.1101/2021.10.29.466444). In our study, the competition matrix was adjusted to reflect moderate drug resistance, allowing clearer demonstration of adaptive therapy efficacy without altering the fundamental competitive relationships.

Reviewer 2

Comment 1:

- **Reviewer's Comment:** While numerical simulations show the superiority of adaptive therapy, it fails to fully eradicate tumors. Moreover, can the game-theoretic model be safely applied to real patients?
- **Authors' Response:** Adaptive therapy aims to maintain a balance between healthy and cancerous cells (sensitive and resistant) to prolong survival, rather than comple te eradication. We acknowledge the need for caution when translating theoretical m odels to clinical practice. However, recent studies (e.g., Virginia et al., https://doi.or g/10.1101/2021.10.29.466444) demonstrate promising clinical validation of game-the oretic models. Future work will integrate more biological mechanisms and deep lea rning to enhance predictive accuracy.

Comment 2:

- **Reviewer's Comment:** A citation is missing in Line 246.
- **Authors' Response:** Thank you for highlighting this. The reference has been added.
- **Revision Location:** Line 247.

We believe these revisions address all reviewers' concerns and significantly enhance the quality of the manuscript. We hope the revised version meets the journal's standards and look forward to your further feedback.

Thank you again for your time and effort.

Sincerely,

Zhiqing Li
Yunnan Minzu University
23213037570002@ymu.edu.cn
February 20, 2025
